# Seroprevalence of *Brucella* infection and associated factors among pregnant women receiving antenatal care around human, wildlife and livestock interface in Ngorongoro ecosystem, Northern Tanzania. A cross-sectional study

**DOI:** 10.1186/s12879-020-4873-7

**Published:** 2020-02-18

**Authors:** Robert Makala, Mtebe V. Majigo, George M. Bwire, Upendo Kibwana, Mariam M. Mirambo, Agricola Joachim

**Affiliations:** 1 Manyara Regional Referral Hospital, Babati, Tanzania; 2 Department of Microbiology and Immunology, Muhimbili University of Health and Allied Sciences, Dar es Salaam, Tanzania; 3 Department of Pharmaceutical Microbiology, School of Pharmacy, Muhimbili University of Health and Allied, Dar es Salaam, Tanzania; 4 Department of Microbiology and Immunology, Weill Bugando School of Medicine, Catholic University of Health and Allied Sciences, Mwanza, Tanzania

**Keywords:** Infection, Pregnancy, Brucellosis, Seroprevalence, Tanzania

## Abstract

**Background:**

Brucellosis is a zoonotic disease transmitted to humans through contact with infected animals, animal products or consumption of infected dairy products. *Brucella* infection during pregnancy is of special interest due to association with adverse pregnancy outcomes. This study determined the seroprevalence and factors associated with *Brucella* infection among pregnant women around the human-wildlife-livestock interface area in Ngorongoro ecosystem, Northern Tanzania.

**Methods:**

A facility-based cross-sectional study was conducted between May and June 2018 at six health facilities that provide antenatal services. Pregnant women receiving antenatal care were invited to participate. A structured questionnaire was used to collect socio-demographic and obstetric characteristics in addition to behavior and practices related to the occurrence of human brucellosis. The presence of serum immunoglobulin against *Brucella* was determined using Rose Bengal Plate Test (RBPT). The positive samples were further assayed for the presence of IgG and IgM using The enzyme-linkedimmunosorbent assay. Bivariate analysis was conducted to determine the variables associated with *Brucella* seropositivity. Multivariable logistic regression analysis was performed to examine the factors independently associations with *Brucella* seropositivity after adjustment for other explanatory variables.

**Results:**

A total of 313 participants were enrolled in the study. The overall seroprevalence of *Brucella* infection was 10.9% (34/313) determined by Rose Bengal plate test. Of 34 positive individuals, 27(79.4%) and 8(23.5%) were positive in the ELISA specific for IgG and IgM *Brucella* antibodies respectively. Regular contact with manure (AOR 3.16, 95%CI 1.27–7.83) and preference for animal fresh milk (AOR 3.80, 95% CI 1.23–11.69), raw meat (AOR 2.58, 95% CI 1.14–5.81) and raw animal blood (AOR 2.71, 95% CI 1.15–6.35) increased the odds of being *Brucella* seropositive. Contact with the animal placenta were not associated with *Brucella* seropositivity after adjustment.

**Conclusion:**

This study has found that brucellosis is an important public health problem among pregnant women in areas with interactions of humans; livestock and wildlife. The risk of infection increased with the regular contact with manure and preference of raw foodstuffs like animal blood, meat, and milk. We emphasize the need for interventional strategies to reduce the risk of exposure.

## Background

Brucellosis is one of the neglected zoonotic diseases, acquired through contact with infected animals, consumption of infected dairy products, or inhalation of aerosols [1, 2]. Wildlife animals near human and domestic animal may act as reservoirs to both [3]. Veterinarians, livestock farmers, milkers, abattoir workers and laboratory workers are occupations at high risk of getting *Brucella* infection [4, 5]. Exposure of wildlife animals to *Brucella abortus* in the Ngorongoro ecosystem has reached 24 and 17% for buffalo and wildebeest populations respectively [6]. The prevalence of brucellosis in domestic ruminants free-range grazing system in Ngorongoro conservation was found to range from 3 to 14.28% in different animals [7].

The community health significance of *Brucella* infection in humans is a severely devastating disease that requires prolonged treatment and may end with disabling results [8]. The major challenge is the similarity of clinical presentation to other febrile illnesses such as malaria and typhoid fever. Consequently, under-reporting and mismanagement may be common in areas with limited laboratory diagnosis [9, 10]. Infection in pregnancy is of major public concerns as it associate with several detrimental pregnancy outcomes like spontaneous abortion, preterm delivery, and fetal death [1, 2, 4, 8]. The risk of low birth weight has been demonstrated to be higher in pregnant women infected with *Brucella* [8]. The major burden of brucellosis is mostly seen in poor individuals living in close contact with animals and having poor access to health care service [11].

Previous studies conducted in Tanzania have reported up to 13% prevalence of brucellosis in the area of pastoral and agro-pastoral communities [11, 12]. However, there is limited published data regarding *Brucella* infections among pregnant women in Tanzania, especially in the area of interactions of humans, livestock and wildlife. This limited information highlights the need to determine the seroprevalence of *Brucella* infection and associated modifiable factors among pregnant women. The information generated from this study may be of help for policy and interventional strategies. Ngorongoro was selected as the study area based on the presence of high interactions among the human-animal-wildlife interface which could play a role in the maintenance of the disease.

## Methods

### Study design and setting

This was a facility-based cross-sectional study conducted between May and June 2018 in Ngorongoro District, Arusha region of Northern Tanzania. The district plays host to parts of the wildebeest migration at the same time cattle, goat and sheep rearing is a common practice. The population of the Ngorongoro District is around 130,000 and the major ethnic groups are the Masai and Sonjo.

The Ngorongoro District has 20 public health facilities including 14 dispensaries, four health centres, and two hospitals. Each of the two hospital records between 25 and 40 new antenatal clinic attendances per week. The study involved six health facilities that provide antenatal services including: Wasso designated district hospital, Sakala and Loliondo health centers as well as Muholo, Sale, and Samunge dispensaries.

### Study population, sample size, and sampling procedure

Pregnant women attending the antenatal clinic at selected health facilities were invited to participate in the study. The study enrolled pregnant women who lived in the study area for more than 3 months and accepting to participate by signing written informed consent were enrolled. The sample size was estimated using Kish Leslie formula [13], at 95% confidence interval (CI) considering 7.7% seroprevalence of *Brucella* infection in Arusha Tanzania [12] and a 3% margin of error. Eligible clients were consecutively enrolled in the study until reaching a *representative* sample size.

### Data collection

A structured questionnaire (Additional file 1) was used to collect the required information from each participant. Data for socio-demographic and obstetric characteristics included: age, marital status, education level, occupation, location, gestation age, gravidity, parity and history of spontaneous abortion. Factors with potential risk for *Brucella* transmission related to animal care, animal product consumption and presence or absence of exposure at the individual level were also collected. The questionnaire included contact with animals and animal products, involvement in milking, sharing water sources with animals, assisting animals to give birth or drink animal fresh milk.

### Study variables

The dependent variable was *Brucella* serostatus as defined using the Rose Bengal Plate Test result and independent variables were behavior and practices with potential risk for *Brucella* infection. Regular contact with animal manure was defined as unprotected exposure to manure at least once in every week in the past 3 months. Participants were counted to contact the placenta if assisted animals giving birth at least once in the past 3 months. Washing animals at home was counted when performed at least once every week for 3 months. Preference of foodstuffs like animal fresh milk, raw animal blood, and raw meat was defined as consumption of the same at least once every week in the past 3 months.

### Specimen collection

Experienced health personnel working at the facilities collected 4 ml of venous blood aseptically using a plain vacutainer system. The collected specimens were labelled with the specific participant’s identification number. Serum samples were separated from whole blood by centrifugation at 3000 rpm for 5 min. The specimens were kept at room temperature for 30 min then at 2–8 °C up to 24 h before processing.

### Laboratory procedure

#### Rose Bengal plate test

The *Brucella* serology was first determined by Rose Bengal Plate Test (RBPT) a rapid agglutination test as previously described [14]. The test does not differentiate antibodies against different *Brucella* species like *Brucella abortus and Brucella melitensis.* Briefly, a drop of serum (50 μl) was taken using a clean micro-pipette onto the test plate beside an equal (50 μl) drop of RBPT antigen. The drops of serum and antigen were mixed using applicator stick then rocked manually for 4 min before examination. The presence of any visible reaction was considered to be positive [15].

#### Enzyme-linked Immunosorbent assay

Positive samples were kept at minus 20 °C before transportation to the reference laboratory in Dar es Salaam for the detection of Immunoglobulin M and G antibodies. The commercially available test kits of enzyme-linked immunosorbent assay (ELISA), SERION ELISA classic *Brucella* IgG/IgM/IgA (Institut Virion/Serion GmbH) was used to detect IgM and IgG antibodies. The technique was performed according to the instructions from the manufacturer. In brief, 100 ml of diluted serum samples and ready to use control were added to the micro test wells containing antigen. The assays were then incubated at 37 °C for 60 min, after which the first wash was performed. Later, anti-human IgM or IgG conjugated with an enzyme was added and incubated for 30 min at 37 °C. All wells were washed to remove excess conjugate, followed by a new incubation for 30 min at 37 °C with the enzyme-substrate. Finally, the reaction was stopped by adding 100 ml of stopping solution. The enzyme reaction with the Substrate yields a coloured product. The colour intensity is proportional to the amount of specific antibody and can be measured by the photometric method.

### Data analysis

Categorical variables were summarized as frequencies and proportions while continuous variables were summarized as median and inter-quartile range (IQR). Group differences were examined using Pearson’s Chi-square test. Bivariate analysis was conducted to determine the variables associated with *Brucella* seropositivity and crude odds ratio (cOR) with 95% CI. Multivariable logistic regression was performed to examine the associations between the outcome variable and independent variables after adjustment for other variables as fixed effect and the cluster variable facility as random effect. Likelihood ratio tests (LRT) was used to simplify the final multivariable model so that only variables that are significantly associated with the outcome are retained in the final model. Associations in the multivariable logistic models were presented as adjusted odds ratios (AOR) with 95% CI. Interactions between independent variables were examined, and the Wald test was used to test the associations of the variables and interactions. The Hosmer-Lemeshow test was used to examine the overall fitness of the model. Statistical Package for Social Sciences version 23 was used for all data analyses. The level of significance was specified at 0.05.

## Results

### Characteristics of participants and seropositivity of *Brucella* infection

A total of 313 participants were enrolled in the study, the median age was 25 years, interquartile range 20–30 years. The majority 299 (95.5%) were Agro-pastoralists, 150 (47.9%) had no formal education, 288 (92.0%) were married, and 201(64.2%) had ≤28 weeks of gestation. Out of 237 with prior pregnancies, 35 (14.8%) reported a history of spontaneous abortion.

All participants were screened for antibodies against y *Brucella* using a rapid RBPT test. Out of 313 participants, 34(10.9% [7.9–14.8]) were seropositive. Of 34 seropositive individuals, 27(79.4%) and 8(23.5%) were positive in the ELISA specific for IgG and IgM antibodies respectively. Based on the detection of IgM antibodies, 2.6% (8/313) of participants were deemed to have had recent *Brucella* infection. The seropositivity observed for demographic (age, occupation, education level, marital status) and obstetric characteristics (gestation age, number of pregnancies, history of spontaneous abortion) were not significantly different (*p* > 0.05) (Table 1).

**Table 1:** Descriptive characteristic of participants and *Brucella* seropositivity based on rapid RBPT

Variable	Frequency	Seropositivity N (%)	*P*-Value*
Overall-seropositivity	313	34 (10.9)	
Age group (years)			
≤ 25	160	16(10.0)	0.616
> 25	153	18(11.8)	
Occupation			
Agro-pastoralist	299	31(10.4)	0.194
Formal employment	14	3(21.4)	
Level of education			
Informal	150	11(7.3)	0.054
Primary	98	11(11.2)	
Secondary and above	65	12 (18.5)	
Marital status			
Single	25	4 (16.0)	0.389
Married	288	30 (10.4)	
Gestation age (weeks)			
≤ 28	201	24 (11. 9)	0.412
> 28	112	10 (8.9)	
Previous pregnancy			
0	76	8(10.5)	
1	76	9(11.8)	
2	58	3 (5.2)	0.421
3+	103	14(13.6)	
Spontaneous abortion (*n* = 237)			
No	202	20(9.9)	0.206
Yes	35	6(17.1)	

*P value according to Pearson Chi-Square test

### Behavior and practice associated with *Brucella* infection

Several factors with the potential risk of brucellosis among humans were assessed and reported in Table 2. Participants who reported to have been exposed to the assessed potential risk factors; had more seropositive cases of *Brucella* except for those reported washing animals at home. At bivariate analysis, regular contact with animal manure increased the probability of *Brucella* seropositivity (cOR 2.7, 95%CI 1.12–6.33). Contact with animal placenta through assisting of parturition had higher odds of being seropositive (cOR 3.1, 95%CI 1.18–8.37). Preference for animal fresh milk, raw meat, and raw animal blood, were significantly associated with seropositivity to *Brucella* (*p* < 0.05). The odds of being seropositive among those prefer animal fresh milk, raw meat and raw animal blood ranged from 2.1 to 3.1 (Table 2). Washing animal at home (*p* = 0.4) and Sharing water source with the animal (*p* = 0.82) were not associated with seropositivity to *Brucella.*

**Table 2:** Bivariate analysis of factors associated with *Brucella* seropositive

Variable	Frequency	Seropositive N (%)	cOR	95%CI	*P*-value
Regular contact with manure					
Yes	192	27 (14.0)	2.7	(1.12–6.33)	**0.022**
No	121	7 (5.8)	1		
Contact with animal placenta					
Yes	210	29 (13.8)	3.1	(1.18–8.37)	**0.017**
No	103	5 (4.9)	1		
Washing animal at home					
Yes	201	20 (10.0)	0.8	(0.37–1.59)	0.487
No	112	14 (12.5)	1		
Preference for fresh milk					
Yes	229	30 (13.1)	3.0	(1.03–8.83)	**0.036**
No	84	4 (4.8)	1		
Preference for raw meat					
Yes	76	13(17.1)	2.1	(1.01–4.48)	**0.044**
No	237	21(8.9)	1		
Preference for raw animal blood					
Yes	174	26 (14.9)	2.9	(1.26–6.57)	**0.009**
No	139	8 (5.8)	1		
Sharing water source with animal					
Yes	160	18 (11.3)	1.1	(0.53–2.21)	0.822
No	153	16 (10.5)	1		

Key: *cOR* Crude odds ratio, *CI* Confidence Interval, *P*-value according to Pearson Chi-Square test

Table 3 shows the result of final model of multivariable regression analysis performed to measure the relationship between *Brucella* seropositivity and independent variables. Variables that showed significant association (*p* < 0.05) in the bivariate analysis were included. Variables with significant LRT were retained in the final multivariable model. The multivariate logistic regression analysis revealed that regular contact with manure, preference for raw animal blood, preference for raw meat, and preference for animal fresh milk remained a risk factor for *Brucella* seropositivity (Table 3). Contact with placenta was not significantly associated with *Brucella* seropositivity after adjustment for other factors (AOR 1.99, 95% CI 0.64–6.28.1, *p* = 0.219).

**Table 3:** Multivariable analysis of factors associated with the RBPT seropositivity of participants

Variable (*P-value)	Seropositive N (%)	AOR	95% CI	**P value
Regular contact with manure (*p* = 0.007)				
Yes	27 (14.0)	3.16	1.27–7.83	0.013
No	7 (5.8)	1		
Preference of raw milk (*p* = 0.008)				
Yes	30 (13.1)	3.80	1.23–11.69	0.020
No	4 (4.8)	1		
Preference of raw meat (*p* = 0.025)				
Yes	26 (14.9)	2.58	1.14–5.81	0.022
No	8 (5.8)	1		
Raw blood consumption (*p* = 0.016)				
Yes	26 (14.9)	2.71	1.15–6.35	0.022
No	8 (5.8)	1		

Key: *AOR* Adjusted odds ratio, *CI* Confidence interval * *P*-value according to Likelihood Ratio Tests, **P-value according to wald test

The significance of odds ratio at 5% level for all variables in the multivariable model did not change after including facility variable to adjust for cluster random effect. Potential interactions between contact with manure and contact with placenta as well as, preference for raw meat and preference for raw animal blood on the outcome of *Brucella* seropositivity were found not significant. Based on these observations the interaction terms were drooped in the final model. The Hosmer-Lemeshow test result was *p =* 0.438 which indicated the fitness of the overall model.

## Discussion

The current study has demonstrated a higher (10.9%, [7.9–14.8]) seropositive of *Brucella* in pregnant women compared to the previous reports in the general population of the same geographical location [12, 16]. Besides, the study has revealed nearly 3% of pregnant women with immunologic evidence of recent *Brucella* infection based on IgM ELISA. The level of seropositive found among pregnant women in the Ngorongoro District suggests that *Brucella* infection is a public health problem. Our finding is higher compared to a previous report from Pakistan (5.8%) among pregnant women [4] but comparable to report from Nepal (11.25%) among pregnant women [17]. Besides, our study finding is lower compared to a report from Uganda (17%) in agro-pastoral communities [18] and 25% among women with abortion in Rwanda [2].

The community where the present study was conducted comprised around 95% agro-pastoralists. In most of the agro-pastoralist communities, women do most of the work associated with care and harvest of livestock products. They actively engage in barn cleaning, herding small ruminants, milking and preparing manure dung [19]. The seroprevalence found in the studied population can be explained by increased risk of exposure due to their routine activities [5]. Brucellosis being an endemic disease in humans and animals [11, 20] and the proximity of population to livestock-wildlife can also contribute to high seroprevalence observed.

The higher seropositivity of *Brucella* infection among pregnant women showed by this study could be attributed in part due to the preference for raw foodstuffs like fresh milk, raw meat, and raw animal blood. Eating habits may expose an individual to *Brucella* infection if the consumed products from infected livestock are not properly prepared [12, 18, 21, 22]. A substantial number of participants in the current study reported a preference for animal fresh milk (73.2%), preference for raw meat (24.3%) and preference for raw animal blood (55.6%). The habits of consumption of raw foodstuff were the risk factors independently associated with *Brucella* infection among pregnant women in Ngorongoro District. Our findings are in agreement with the previous study conducted in Tanzania which reported food preferences and eating behavior to play major roles in *Brucella* infection in pastoral and agro-pastoral communities [5, 12]. Other studies in Africa also reported similar predictors for transmission of brucellosis, although the main predictors vary depending on customs and taboos of referred community [23, 24].

Regular contact with animal manure and contact with the placenta had increased odds of being seropositive for *Brucella* infection in the bivariate analysis model. However, contact with animal placenta was not associated with *Brucella* seropositivity in the multivariable logistic regression model. Some studies also reported direct contact with livestock excreta as a potential route of exposure to *Brucella* infection [12, 23]. Similarly, contact with animal placenta has been reported associated with brucellosis [25–28]. It is also documented that *Brucella* spp. from infected animals are found in animal excreta which serve as sources of humans infections [29]. There was no evidence from the analysis to explain the lack of independent association of *Brucella* infection with exposure to animal placenta. The potential interaction with other variables was not supported by the multivariable logistic regression model.

The study relied heavily upon self-reported information which is open to information bias, clustering of events and failure to recall. Participants could have missed out on some possible factors associated with the occurrence of brucellosis. Reporting error for some measures was reduced by asking participants to recall only events in the last 3 months. Despite the limitations encountered, this study has demonstrated some important factors associated with transmission of *Brucella* to humans in the Ngorongoro ecosystem. Our findings serve as considerable baseline data for prevention and control of the disease and associated adverse effect in pregnancy.

## Conclusion

This study has found that brucellosis is an important public health problem among pregnant women in the area with interactions of humans; livestock and wildlife. The risk of infection increased with the preference of raw foodstuffs like animal blood, meat, and milk. These findings emphasize the need for interventional strategies to reduce the risk of exposure and improve early detection of infection in pregnant women.

## Supplementary information

**Additional file 1.** Interview questionnaire.

## Data Availability

The datasets used and analysed for the current study are available from the corresponding author on reasonable request.

## Abbreviations

ELISA: Enzyme-linked immunosorbent assay
IgG: Immunoglobulin G
IgM: Immunoglobulin M
MUHAS: Muhimbili University of Health and Allied Sciences
RBPT: Rose Bengal plate test
