# Supplementary material for: Seroprevalence of Brucella infection and associated factors among pregnant women receiving antenatal care around human, wildlife and livestock interface in Ngorongoro ecosystem, Northern Tanzania. A cross-sectional study
Source: BMC Infect Dis. 2020 Feb 18;20:152. doi: 10.1186/s12879-020-4873-7 (PMC7029502; doi:10.1186/s12879-020-4873-7)
Supplement: Supplementary file 1 — Additional file 1. Interview questionnaire. [file 12879_2020_4873_MOESM1_ESM.doc]

**INTERVIEW QUESTIONNAIRE**

**Sero-prevalence of *Brucella* infection among Pregnant Women Attending Antenatal Clinic around Human-Wildlife-Livestock Interface Area in Ngorongoro Ecosystem, Northern Tanzania**

**Identification number [______________________]**

*“I’d like to thank you for taking part in this interview. All the information you give me will be confidential and your name won’t be recorded anywhere. The answers to some questions may seem obvious to you, but I need to ask you all of the questions. You are free to end this interview session at any point if you feel uncomfortable”*

| **General Information** | | | | | | | | | | | | |  |
| --- | --- | --- | --- | --- | --- | --- | --- | --- | --- | --- | --- | --- | --- |
| Location and Date | | | Response | | | | | | | Code | | |  |
| Name of Facility | | |  | | | | | | |  | | |  |
| Name of village/Street |  | | | | | |  | | | |  | | |
| Name of Ward |  | | | | | |  | | | |  | | |
| Interviewer ID | └─┴─┴─┘ | | | | | |  | | | |  | | |
| Date of the interview | └─┴─┘ └─┴─┘ └─┴─┴─┴─┘  dd mm year | | | | | |  | | | |  | | |
| Consent, Interview Language and Name | | | Response | | | | | | | Code | | |  |
| Consent has been read and obtained | | | | Yes | 1 | | |  | | | | | |
|  | | | | No | 2 If NO, END | | |  | | | | | |
| Interview Language | | | | English | 1 | | |  | | | | | |
|  |  |  |  | Kiswahili | 2 | | |  | | | | | |
|  |  |  |  | Others | 3*[Insert Language]* | | |  | | | | | |
| Time of interview (24 hour clock) | | | └─┴─┘: └─┴─┘  hrs min | | | | | | |  | | |  |
| **Participant Demographic Information** | | | | | | | | | | | | |  |
| **Question** | | | **Response** | | | | | | | **Code** | | |  |
| 1. What is your date of birth? *Don't Know 77* | | | └─┴─┘ └─┴─┘ └─┴─┴─┴─┘  dd mm year | | | | | | |  | | |  |
| 2. How old are you? | | | Years | | | └─┴─┘ | | | |  | | |  |
| 3. In total, how many years have you spent at school and in full-time study (excluding pre-school)? | | | Years└─┴─┘ | | |  | | | |  | | |  |
| 4. What is the highest level of education you have completed? | | | No formal schooling | | | 1 | | | |  | | |  |
|  |  |  | Less than primary school | | | 2 | | | |  |  |  |  |
|  |  |  | Primary school | | | 3 | | | |  |  |  |  |
|  |  |  | Secondary school | | | 4 | | | |  |  |  |  |
|  |  |  | High school | | | 5 | | | |  |  |  |  |
|  |  |  | College/University | | | 6 | | | |  |  |  |  |
|  |  |  | Post graduate degree | | | 7 | | | |  |  |  |  |
|  |  |  | Refused | | | 88 | | | |  |  |  |  |
|  | | | Don’t Know | | | 77 | | | |  | | |  |
| 5. What is your marital status? | | | Never married | | | 1 | | | |  | | |  |
|  |  |  | married | | | 2 | | | |  |  |  |  |
|  |  |  | Separated | | | 3 | | | |  |  |  |  |
|  |  |  | Widowed | | | 4 | | | |  |  |  |  |
|  |  |  | Cohabitating | | | 5 | | | |  |  |  |  |
|  |  |  | Refused | | | 88 | | | |  |  |  |  |
| 6. Which of the following best describes your main work status over the past 12 months? | | | Government employee | | | 1 | | | |  | | |  |
|  |  |  | Non-government employee | | | 2 | | | |  |  |  |  |
|  |  |  | Self-employed | | | 3 | | | |  |  |  |  |
|  |  |  | Peasant | | | 4 | | | |  |  |  |  |
|  |  |  | House wife | | | 6 | | | |  |  |  |  |
|  |  |  | Farmer | | | 7 | | | |  |  |  |  |
|  |  |  | Refused | | | 88 | | | |  |  |  |  |
| **Obstetric Information** | | | | | | | | | | | |  | |
| 7. What is the gestation age of the current pregnancy? (weeks)________ | | | | | | | | |  | | |  | |
| 8. Including the current pregnancy, how many pregnancies have you ever had? (*99 if not applicable)* | | | | | | | | |  | | |  | |
| 9. If you had previous pregnancies; how many pregnancies ended as abortion? (*99 if not applicable)* | | | | | | | | |  | | |  | |
| 10. If you had abortion, how many were spontaneous abortion? (*99 if not applicable)* | | | | | | | | |  | | |  | |
| 11. Tell me the number of babies born before full term gestation ((*99 if not applicable)* | | | | | | | | |  | | |  | |
| **Behavior and practice** | | | | | | | | | | | |  | |
| 12. Do you prefer consumption of animal raw milk? | | | Yes | 1 | | --- | --- | | No | 2 | | Refused | 88 | | | | | | | |  | | |  | |
| 13. If you prefer raw milk, how frequent did you take animal raw milk in the past three months? | | | Daily | 1 | | --- | --- | | At least once per week | 2 | | At least once per month | 3 | | Refused | 88 | | Not applicable | 99 | | | | | | | |  | | |  | |
| 14. Do you prefer consumption of raw meat? | | | Yes | 1 | | --- | --- | | No | 2 | | Refused | 88 | | | | | | | |  | | |  | |
| 15. If you prefer raw meat, how frequent did you eat raw meat in the past three months? | | | Daily | 1 | | --- | --- | | At least once per week | 2 | | At least once per month | 3 | | Refused | 88 | | Not applicable | 99 | | | | | | | |  | | |  | |
| 16. Do you prefer consumption of animal fresh blood? | | | Yes | 1 | | --- | --- | | No | 2 | | Refused | 88 | | | | | | | |  | | |  | |
| 17. If you prefer animal fresh blood, how frequent did you take animal fresh blood in the past three months? | | | Daily | 1 | | --- | --- | | At least once per week | 2 | | At least once per month | 3 | | Refused | 88 | | Not applicable | 99 | | | | | | | |  | | |  | |
| 18. What is the usual source of water for domestic use? | | | Tape water | 1 | | --- | --- | | Dam | 2 | | River | 3 | | Borehole | 4 | | | | | | | |  | | |  | |
| 19. Do you normally share the same water source with animals? | | | Yes | 1 | | --- | --- | | No | 2 | | Refused | 88 | | Not applicable | 99 | | | | | | | |  | | |  | |
| 20. Have you ever washed animal at home? | | | Yes | 1 | | --- | --- | | No | 2 | | Do not remember | 3 | | Refused | 88 | | | | | | | |  | | |  | |
| 21. If No. 20 is yes; In the past three months, how frequent did you wash animal at home? | | | Daily | 1 | | --- | --- | | At least once per week | 2 | | At least once per month | 3 | | Refused | 88 | | Not applicable | 99 | | | | | | | |  | | |  | |
| 22. Have you ever slaughtered animal at home? | | | Yes | 1 | | --- | --- | | No | 2 | | Do not remember | 3 | | Refused | 88 | | | | | | | |  | | |  | |
| 23. If No. 22 is yes; In the past three months, how frequent did slaughter animal at home? | | | Daily | 1 | | --- | --- | | At least once per week | 2 | | At least once per month | 3 | | Refused | 88 | | Not applicable | 99 | | | | | | | |  | | |  | |
| 24. Have you ever clean animal structure by removing animal manure? | | | Yes | 1 | | --- | --- | | No | 2 | | Do not remember | 3 | | Refused | 88 | | | | | | | |  | | |  | |
| 25. If No. 24 is yes; In the past three months, how frequent did you remove animal manure from animal structure without protection? | | | Daily | 1 | | --- | --- | | At least once per week | 2 | | At least once per month | 3 | | Refused | 88 | | Not applicable | 99 | | | | | | | |  | | |  | |
| 26. Have you ever assisted animal during parturition? | | | Yes | 1 | | --- | --- | | No | 2 | | Do not remember | 3 | | Refused | 88 | | Not applicable | 99 | | | | | | | |  | | |  | |
| 27. If you have assisted animal during parturition, how frequent you assisted in the last three months? | | | Daily | 1 | | --- | --- | | At least once per week | 2 | | At least once per month | 3 | | Refused | 88 | | Not applicable | 99 | | | | | | | |  | | |  | |
